# From Mechanisms to Commercialization: CuO Positive Electrodes for Rechargeable Alkaline Zinc Batteries

**DOI:** 10.1021/acsomega.6c04389

**Published:** 2026-08-27

**Authors:** Yogeshwaran Agilan, Bryan R. Wygant, Timothy N. Lambert, Joshua W. Gallaway

**Affiliations:** † Department of Chemical Engineering, 1848 Northeastern University, 360 Huntington Avenue, Boston, Massachusetts 02115, United States; ‡ Nanoscale Sciences Department, 1105 Sandia National Laboratories, Albuquerque, New Mexico 87185, United States of America; § Department of Photovoltaics and Materials Technology, Sandia National Laboratories, PO Box 5800, Albuquerque, New Mexico 87185, United States; ∥ Center for Integrated Nanotechnologies, Sandia National Laboratories, PO Box 5800, Albuquerque, New Mexico 87185, United States

## Abstract

Cupric oxide (CuO) is a promising positive electrode material for rechargeable alkaline Zn batteries, offering earth-abundant composition, high theoretical capacity (674 mAh/g), and compatibility with safe aqueous electrolytes. Despite early commercial success as a primary battery dating to the Lalande-Chaperon cell (1883), rechargeability has only recently been demonstrated with Bi_2_O_3_ additives and carbon coatings, which help suppress resistive Cu_2_O accumulation and limit active material dissolution. Significant barriers remain, including poorly understood failure mechanisms at high depth of discharge and limited cathode utilization. This review summarizes the electrochemical mechanisms of Cu and CuO in alkaline electrolyte, critically examines recent advances in Bi_2_O_3_-modified and carbon-coated electrodes, including uncertainty about charge products and the mechanistic role of Bi, and identifies priority directions for future research. A cell-level energy density and cost analysis demonstrates that cathode areal capacity and Zn anode utilization are the dominant cost drivers, with areal capacities exceeding 40 mAh/cm^2^ required to reach storage costs below 100 USD/kWh, a target achievable with existing materials if electrode engineering challenges are resolved.

## Graphical abstract

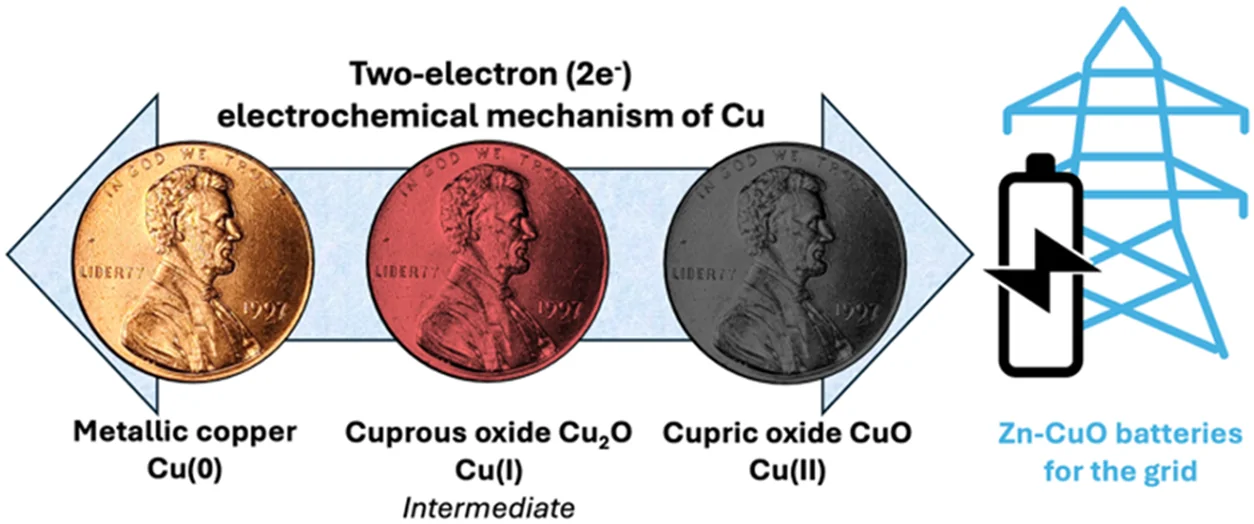

## 1 Introduction

Batteries are ideal candidates for large-scale storage applications as they store electrical energy at lower installation cost than alternatives such as pumped hydro, flywheels, and capacitors.^1^ They provide rapid response to load changes and enable voltage and frequency regulation.^2^ For large-scale applications, safety, cost, and cycle life are of greater concern than energy and power density.^3,4^ Li-ion batteries are currently deployed for the grid due to their high efficiency, long cycle life, and high energy density,^5^ but they carry serious safety risks from flammable organic electrolytes.^6^ While Li-ion costs continue to fall, resource limitations and elevated costs of Li, Co, and Ni—major components in various Li-ion configurations—remain concerns.^3,7^

The elemental costs of several battery-relevant materials are listed in Table 1. Zn, Mn, and Cu fall in the 1–10 USD/kg range, indicating a low material cost basis for large-scale deployment. Cu has historically been in this range but is currently elevated due to market uncertainty and other factors. However, there are indications that the 2022–2024 level of ∼8.50–9.30 USD/kg is a suitable assumption for long-term cost projections. Co and Ni, which are important for layered oxide type Li-ion batteries, range from 13–68 USD/kg, increasing capital expenditure. It should be noted that elemental prices are approximations for cost effects in practical batteries: for example, the raw material for production of lithium iron phosphate (LFP or LiFePO_4_) is often battery grade phosphoric acid or H_3_PO_4_, which is ∼1/3 the cost of elemental white phosphorus.^8^ However, this comparison does convey the fundamental cost penalty of certain materials for use in low-cost batteries.

**Table 1:** Elemental Raw Material Prices Collected from Commodity Market Sources at the Time of Writing (July 2026): Fe = Iron Ore Commodity Indices; Mn = IMARC Group; P = Intratec White Phosphorus; Zn, Cu, Ni, Co = London Metal Exchange (LME)^a^

Element	Current (Jul 2026) USD/kg	2022–2024 annual avg range USD/kg	Notes on volatility
Fe	∼0.10	∼0.09–0.13	stable; iron ore ∼100 USD/t is typical
Mn	∼1.31	∼0.95–1.31	relatively stable; steel-driven demand
Zn	∼3.55	∼2.90–3.60	moderate volatility
P	∼4.80	∼3.00–5.00	US price; china ∼3.20 USD/kg currently
Cu	∼13.60	∼8.50–9.30	currently elevated**;** 2022–2024 LME avg was ∼8.5–9.3 USD/kg
Ni	∼16.35	∼13–24	peaked ∼24 USD/kg in 2022, has since declined
Co	∼56.29	∼26–68	highly volatile; 2024 avg was ∼26 USD/kg (LME); 2022 peak ∼68 USD/kg

a Historical range column draws on USGS Mineral Commodity Summaries. Prices in US dollars (USD).

Recent work has focused on the electrochemistry of oxides based on low-cost first-row transition metals such as MnO_2_.^9,10^ Alkaline CuO cathodes are among the best candidates for large-scale storage, as they use earth-abundant materials, employ water-based electrolytes for inherent safety, and exhibit a high theoretical capacity (674 mAh/g).^11^ CuO can be paired with an alkaline Zn anode, which is also earth-abundant, compatible with aqueous electrolytes, and has a high theoretical capacity (820 mAh/g).^12^

Felix Lalande and Georges Chaperon patented the primary Zn-CuO battery in 1883 (the Lalande-Chaperon cell),^13^ which Thomas Edison further developed into the Edison-Lalande cell. These primary cells shared the same electrochemistry. The difference involved electrode fabrication: the Lalande-Chaperon cell employed loose powdered CuO as the cathode material, while Edison’s advance was compressing the CuO into solid briquettes, improving mechanical integrity and handling.^14^ Cells had a low voltage of 0.75 V but also low internal resistance, allowing high discharge current.^11,14^ These were distinct from the primary Zn–Cu Daniell cell (∼1 V, Cu metal cathode, divided CuSO_4_|ZnSO_4_ electrolytes). CuO has not been used in secondary batteries due to the formation of resistive Cu_2_O intermediates that hinder rechargeability.^15^

Recent advancements have demonstrated rechargeability of CuO electrodes through modification with a small quantity of bismuth oxide, which decreases electrode resistance and enables cycling at limited depth of discharge (DOD).^11^ However, challenges remain at higher DOD.^16^ This review covers the status of alkaline Zn-CuO batteries and the challenges hindering their use in commercial grid-scale energy storage, as summarized in Figure 1.

**Figure 1:**
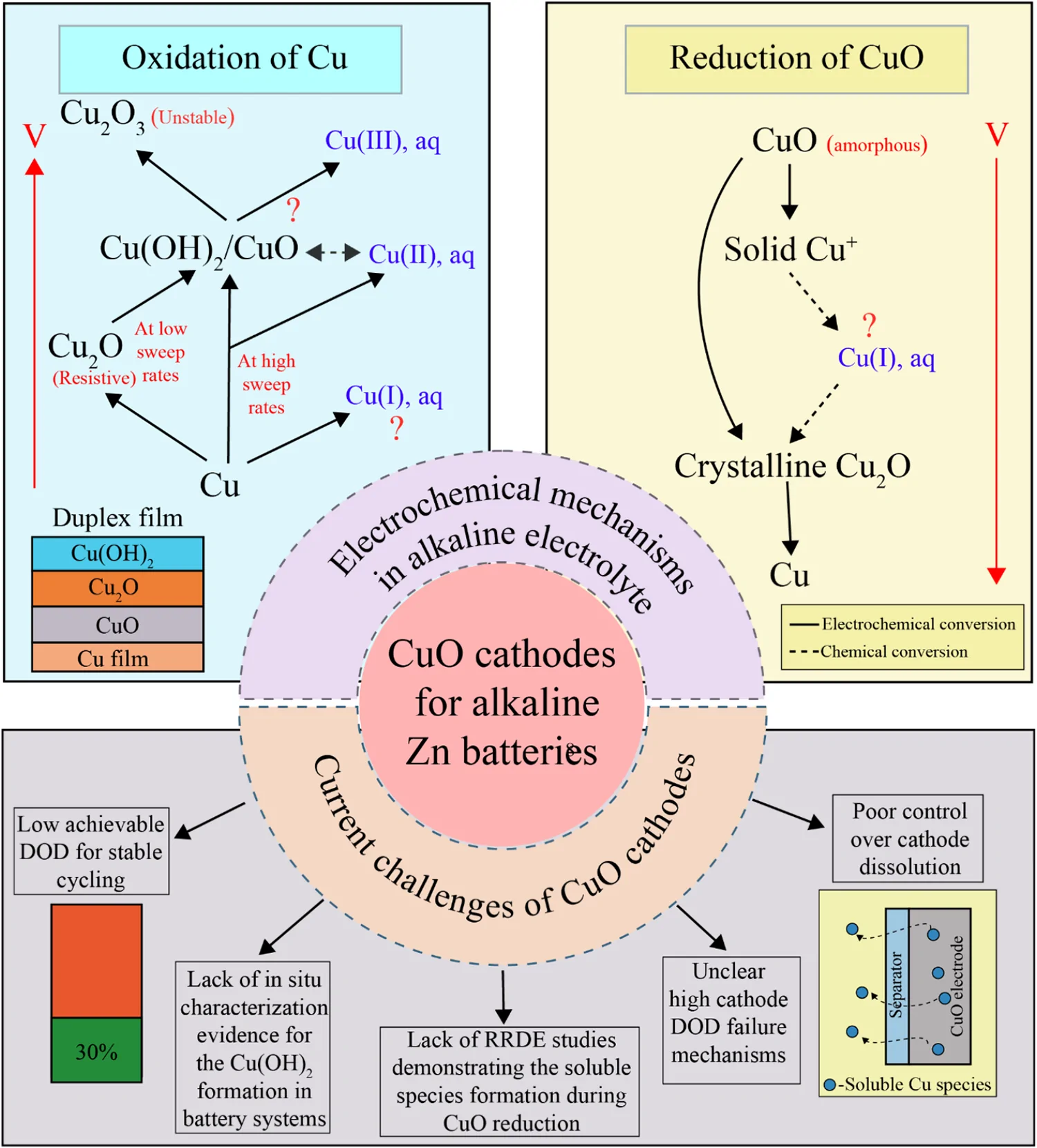
Electrochemical mechanisms of CuO cathodes in alkaline electrolyte and current research challenges. Mechanistically, electrochemical conversions are marked by solid lines, while chemical conversions are dashed lines.

## 2 CuO Cathode Materials

### 2.1 Historical Zn-CuO Batteries

Alkaline Zn-CuO batteries saw several early commercial applications. Among the earliest electrically powered submarines, *Gymnote* (launched 1888) was the first vessel capable of carrying torpedoes, originally propelled by Lalande-Chaperon cells.^18^ An Edison-Lalande primary cell is shown in Figure 2a, with the central Cu/CuO plate sandwiched by Zn plates on either side.^14^ Such wet cells were used when large capacity was needed with high currents at nearly constant voltage, including “mine, highway, railway, and marine signaling installations, for telephone and telegraph circuits,” either as the direct power source or as standby.^17^ By 1952, more than 1.5 million Lalande cells were produced annually in the US. The 500 Ah railway signal cell shown in Figure 2b used a five-electrode assembly (three anodes, two cathodes), with an initial open circuit voltage (OCV) of 1.06 V prior to discharge and a normal operating discharge voltage of 0.5–0.7 V (Figure 2c).

**Figure 2:**
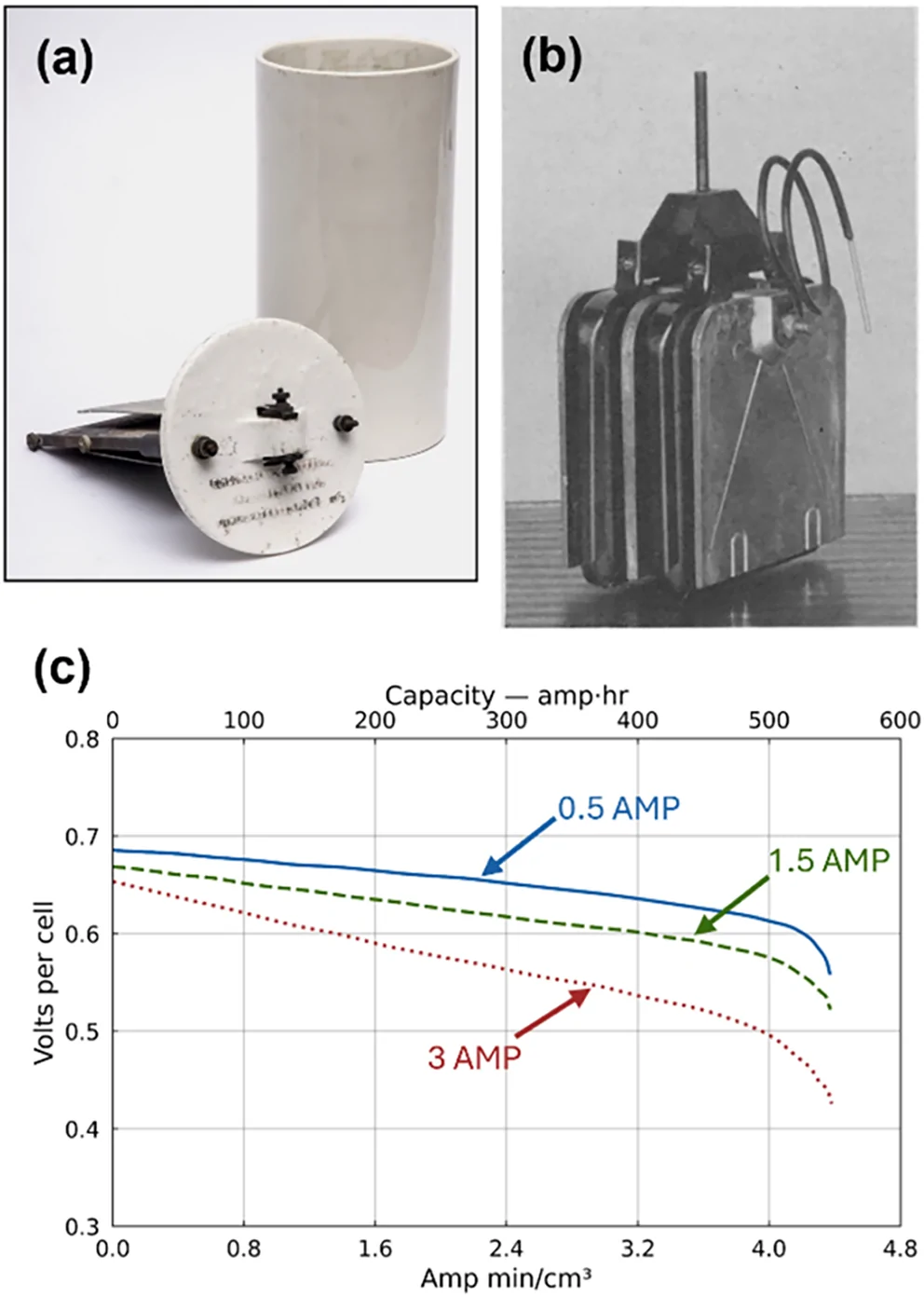
Early Zn-CuO primary cells. (a) Edison-Lalande primary cell. Reused with the permission of the Oesper Collections in the History of Chemistry, University of Cincinnati Libraries.^14^ (b) Five-electrode assembly for 500 Ah Zn-CuO railway signal cell.^17^ (c) Discharge voltage profile of five-electrode assembly as shown in (b).^17^ (b, c) Reproduced and adapted respectively from ref 17 with permission. ©The Electrochemical Society. Reproduced by permission of IOP Publishing Ltd. All rights reserved.

### 2.2 The Electrochemistry of Cu in Alkaline Electrolyte

#### 2.2.1 Oxidation of Copper Metal

The oxidation of Cu metal in alkaline electrolyte involves multiple solid species of differing morphologies as well as dissolved species, with the primary species being Cu, Cu_2_O, CuO, Cu­(OH)_2_, Cu_2_O_3_, and [Cu­(OH)_4_]^2–^, as illustrated in the electrochemical mechanism summary in Figure 1. Voltammetric studies of Cu metal have provided much mechanistic information.

Giri and Sarkar showed a typical cyclic volammogram (CV) of Cu metal in alkaline electrolyte (Figure 3a),^19^ finding that Cu oxidation proceeds through a multistep electrochemical process whose product distribution is strongly influenced by scan rate and electrolyte concentration. At high scan rates (50 mV/s), Cu is oxidized to Cu_2_O via reaction ([Disp-formula eq1]), as indicated by peak A. It is important to note that the amount of Cu_2_O generated, as evident from the capacity of peak A, was relatively small. Increasing potential further oxidized Cu to [Cu­(OH)_4_]^2–^ and Cu­(OH)_2_ via reactions ([Disp-formula eq2]) and ([Disp-formula eq3]), corresponding to peak B1. The large capacity of peak B1 demonstrates that the reaction is largely not sequential, with metallic Cu being the majority reactant in reactions ([Disp-formula eq1]–[Disp-formula eq3]). A further increase in potential produced CuO and Cu­(OH)_2_ simultaneously via reactions ([Disp-formula eq4]) and ([Disp-formula eq3]), attributed to peak B2.^19^ Thus, during oxidation of planar Cu metal, the oxidation intermediate Cu_2_O is mainly observed as a thin film that initially forms on the surface, and reactions ([Disp-formula eq2]–[Disp-formula eq4]) proceed through this film. At lower scan rates (10 and 20 mV/s), the peak current for Cu_2_O formation increased ∼3x, suggesting enhanced diffusion of OH^–^ ions through the Cu_2_O film and more complete oxidation of the underlying Cu metal. At lower scan rate, some reactions for Cu_2_O oxidation to Cu­(II) species were also reported, suggesting that sequential reaction through Cu(0) → Cu_2_O → Cu­(II) occurs to a greater extent at slow scan rate.

1 $$2{\mathrm{Cu}}^{0}+2{\mathrm{OH}}^{-}⇌{{\mathrm{Cu}}^{I}}_{2}O+H_{2}O+2e^{-}$$

2 $${\mathrm{Cu}}^{0}+4{\mathrm{OH}}^{-}⇌{\left[{\mathrm{Cu}}^{\mathrm{II}}{\left(\mathrm{OH}\right)}_{4}\right]}^{2-}+2e^{-}$$

3 $${\mathrm{Cu}}^{0}+2{\mathrm{OH}}^{-}⇌{\mathrm{Cu}}^{\mathrm{II}}{\left(\mathrm{OH}\right)}_{2}+2e^{-}$$

4 $${\mathrm{Cu}}^{0}+2{\mathrm{OH}}^{-}⇌{\mathrm{Cu}}^{\mathrm{II}}O+H_{2}O+2e^{-}$$

The electrochemical reactions detailed in ([Disp-formula eq1]–[Disp-formula eq4]) provide the majority reactions producing the chief product species, and these are also the ones labeled in Figure 3a. The Giri and Sarkar reference also provides reactions for intermediate species along with the effects of scan rate and KOH concentration, and readers seeking more detail are directed to this work. Of primary concern for this review, the oxidation of Cu metal in alkaline electrolyte is demonstrated to be multistep, with the peak B reactions being significantly more complicated than those of peak A. Cu metal is often the source of Cu­(II) species, with Cu_2_O largely being observed as a film, which is discussed in more detail below. Because Cu oxidation in rechargeable batteries will proceed from particles instead of planar Cu foil, the effect of morphology on the reaction sequences observed in (1–4) is of interest for further research.

**Figure 3:**
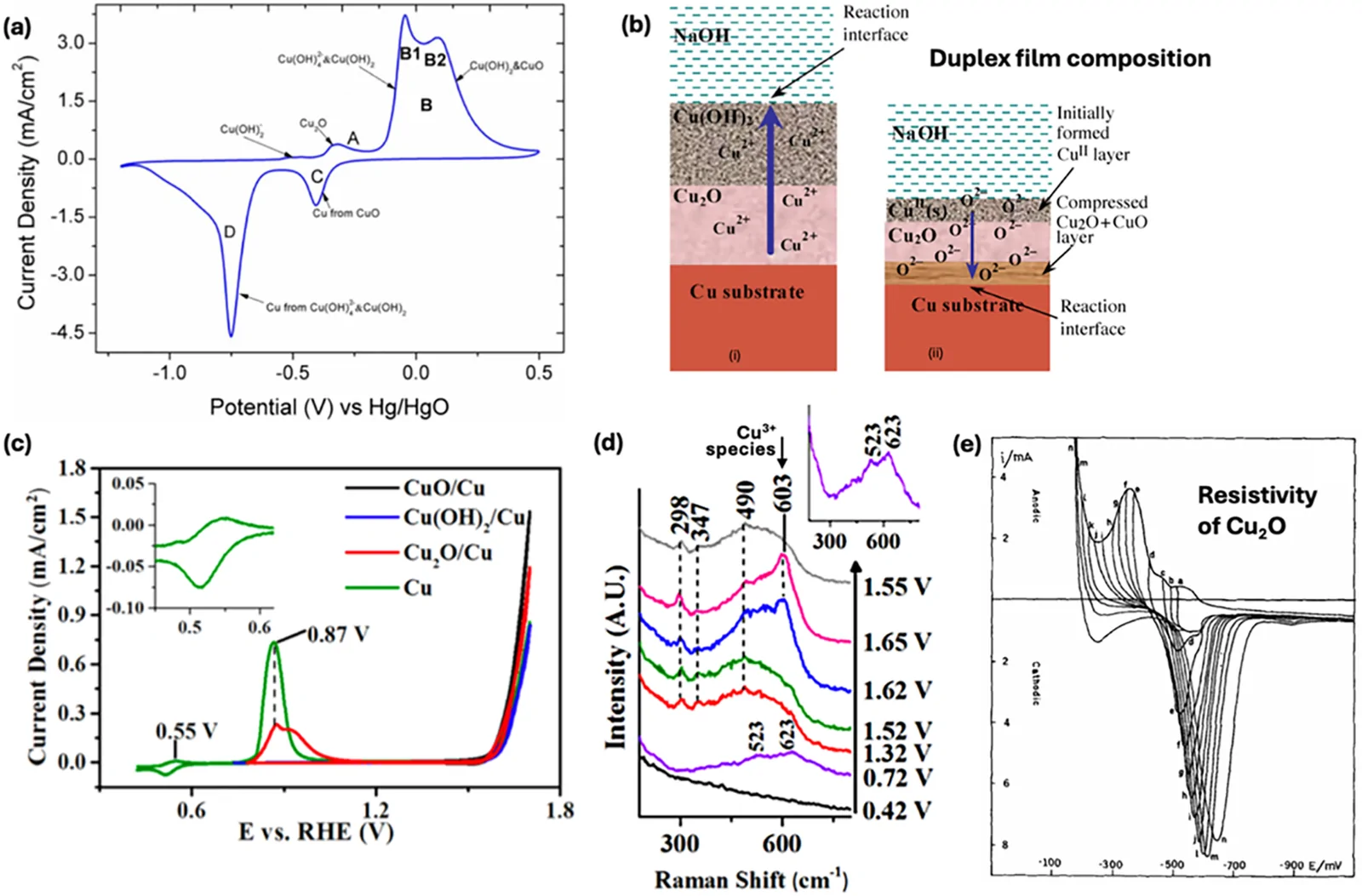
Studies of Cu metal cyclic voltammetry (CV) in alkaline electrolyte. (a) CV at a scan rate of 50 mV/s in 0.5 M KOH solution. Reproduced from ref 19 under CC BY 4.0. (b) Schematic cross-section of the duplex film structure and ion transfer processes within the film formed during (i) partial passivation region, and (ii) final passivation region. [Reprinted from *Applied Surface Science*, vol. 253, He et al., “Potential dependence of cuprous/cupric duplex film growth on copper electrode in alkaline media”, pp. 689–697, Copyright (2006), with permission from Elsevier.][Bibr ref20] (c) CV of various Cu films at 1 mV/s in 0.1 M KOH solution. (d) In situ Raman spectra during CV of Cu in panel c. [(c, d) Reprinted with permission from Deng et al., ACS Catalysis, 6, 2473 (2016). Copyright 2016 American Chemical Society.][Bibr ref21] (e) CV of metallic copper at 0.1 V/s in 1 M KOH solution with increasing sweep range. [Reprinted from *Journal of Electroanalytical Chemistry and Interfacial Electrochemistry*, vol. 47, Ambrose et al., “Investigations of copper in aqueous alkaline solutions by cyclic voltammetry”, pp. 47–64, Copyright (1973), with permission from Elsevier.][Bibr ref22]

The CV results of Giri and Sarkar represent the most recent full study of Cu oxidation, building on the work of many others. The paragraphs following are historical, providing an overview of some of the key work leading up to the Giri and Sarkar’s modern study, as well as more detailed studies of surface film composition. In 1973, Ambrose and co-workers reported the CV of a Cu electrode in 1 M KOH, noting several peaks and the formation of a complex surface film during oxidation^22^—later termed the duplex film. In 1976, Shoesmith et al. reported that CuO was the dominant Cu­(II) species at low current densities, while Cu­(OH)_2_ predominated at higher current densities.^23^ In 1991, Schwartz and Muller used Raman spectroscopy to characterize the duplex film,^24^ observing initial Cu_2_O formation covered by porous Cu­(OH)_2_, followed by further Cu_2_O oxidation to form a CuO underlayer coexisting with the Cu­(OH)_2_ upper layer. This structure of the duplex film is illustrated in Figure 3. They also noted that film reduction was affected by illumination, attributed to photogenerated charge carriers that decreased film resistance.^15,24^

In 2006, He and co-workers^20^ proposed a field-assisted ion transfer mechanism for duplex film formation (Figure 3b). Initially, the Cu metal oxidized to Cu_2_O. Upon further potential increase, Cu­(OH)_2_ formed at the electrolyte interface via Cu^2+^ transport through the Cu_2_O layer. At higher potentials (above 0.18 V vs Hg|HgO), a CuO layer formed beneath the Cu_2_O layer via O^2–^ migration. He and co-workers used X-ray photoelectron spectroscopy (XPS) to differentiate CuO, Cu_2_O, and Cu­(OH)_2_ by binding energies and shakeup peaks, combined with chronopotentiometry and capacity measurements to determine film composition and thickness.

Several studies have suggested a metastable Cu­(III) species at elevated potentials. Miller et al.^25^ used rotating ring disk electrode (RRDE) to demonstrate unstable Cu­(III) formation at high anodic potentials. Deng et al.^21^ prepared films of Cu_2_O, CuO, and Cu­(OH)_2_ on copper substrates to investigate their oxygen evolution reaction (OER) behavior using CV, chronoamperometry, and in situ Raman spectroscopy in 0.1 M KOH. All four copper samples oxidized to CuO and Cu­(OH)_2_ and catalyzed OER with similar efficiencies above 1.62 V vs reversible hydrogen electrode (RHE) (Figure 3c), with Cu­(OH)_2_ and CuO confirmed by Raman bands at 488 and 298 cm^–1^, respectively. A “CuO_2_^–^-like” Cu­(III) oxide appeared near the OER onset with a Raman peak at 603 cm^–1^ (Figure 3d). Mayer et al.^15^ assigned a broad band at 550 cm^–1^ to a Cu­(III) species, likely Cu_2_O_3_, consistent with several other studies.^26–28^

The resistive nature of the Cu_2_O film was demonstrated by Ambrose et al.,^22^ who progressively increased the CV sweep range while holding the upper limit at the Cu_2_O anodic peak. Peak height increased with sweep range, indicating greater Cu_2_O formation during the anodic sweep, while the cathodic peak shifted to more negative potentials, reflecting increasing overpotential for reduction of the passivating Cu_2_O film.

Results above demonstrate that Cu oxidation does not proceed as a simple two-step reaction, and many Cu­(II) species are products of direct oxidation from Cu(0). For rechargeable batteries, this raises the critical question of how the duplex film mechanism is altered in a particulate system, and how the mechanism responds to, for example, changes in Cu particle size. The growing oxide film acts as the primary rate-controlling barrier to further oxidation, as continued oxidation requires ionic species to diffuse through the existing film to reach the reaction interface.^29^ Particle size therefore directly governs the oxide film thickness and the ionic diffusion length, with smaller particles reaching complete oxidation more rapidly due to shorter diffusion distances.

#### 2.2.2 Reduction of CuO in Alkaline Electrolyte

In the previous section, oxidation of Cu metal was reviewed, which represents the charge reaction in batteries. The reduction of CuO is the battery discharge mechanism and must also be well understood. In Giri and Sarkar’s CV at high scan rate (50 mV/s), the first cathodic peak (peak C, Figure 3a) was attributed to reduction of CuO to Cu metal, driven by a high thermodynamic driving force.^19^ A further cathodic shift produced a second reduction peak (peak D) attributed to reduction of Cu­(OH)_2_ and [Cu­(OH)_4_]^2–^ to Cu metal. However, the active material in alkaline batteries is typically not formed from Cu foil, as devices are assembled in the charged state. Instead, the active material is typically CuO particles forming a pasted electrode with a conductive and porous network. This fabrication method alters electrochemical behavior relative to Cu substrates, as oxide overlayers inhibit surface reactions of the underlying Cu.

Mechanistic studies of CuO have been important in understanding cathode discharge. In 2011, Jones et al.^32^ studied amorphous CuO for high-rate discharge in primary alkaline batteries, reporting better performance than electrolytic manganese dioxide (EMD). In 2013, Jones et al.^30^ prepared two CuO types: amorphous CuO via room-temperature precipitation (glycol-CuO) and highly crystalline CuO via high-temperature solid-state decomposition of Cu­(NO_3_)_2_·2.5H_2_O at 450 °C for 24 h (thermal-CuO). Linear sweep voltammetry (LSV) of both materials in 9 M KOH at 0.02 mV/s is shown in Figure 4a. Crystalline CuO underwent single-step reduction to Cu via reaction ([Disp-formula eq5]). Amorphous CuO followed a two-step process: first, a dissolution–precipitation mechanism to Cu_2_O via reaction ([Disp-formula eq6]) with soluble Cu­(I) as the intermediate (Figure 4b), then reduction of Cu_2_O to Cu via reaction ([Disp-formula eq7]). This two-step reduction was further supported by Nakayama et al.^33^

5 $${\mathrm{CuO}}^{\left(\mathrm{II}\right)}+H_{2}O+2e^{-}⇌{\mathrm{Cu}}^{\left(0\right)}+2{\mathrm{OH}}^{-}$$

6 $$2{\mathrm{CuO}}^{\left(\mathrm{II}\right)}+H_{2}O+2e^{-}⇌{\mathrm{Cu}}_{2}O^{\left(I\right)}+2{\mathrm{OH}}^{-}$$

7 $${\mathrm{Cu}}_{2}O^{\left(I\right)}+H_{2}O+2e^{-}⇌2{\mathrm{Cu}}^{\left(0\right)}+2{\mathrm{OH}}^{-}$$

Jones et al.^30^ proposed two possible mechanisms—ECC (Electrochemical-Chemical-Crystallization) and CEC (Chemical-Electrochemical-Crystallization)—for CuO-to-Cu_2_O conversion during reduction in 9 M KOH (Figure 4b). In the ECC mechanism, an initial electrochemical step inserts an electron into solid CuO, reducing Cu^2+^ to Cu^+^ within the solid, followed by chemical dissolution to soluble Cu­(I) and chemical precipitation as crystalline Cu_2_O. In the CEC mechanism, CuO is first chemically dissolved as [Cu­(OH)_4_]^2–^, then electrochemically reduced to soluble Cu­(I), and finally precipitated as Cu_2_O. By comparing the voltammetry of solid CuO versus dissolved [Cu­(OH)_4_]^2–^ in 9 M KOH and observing different reduction potentials, they concluded that discharge follows the ECC mechanism. This was supported by the voltage profile (Figure 4c), as a potential dip between the first plateau (CuO→Cu_2_O) and second plateau (Cu_2_O→Cu) was attributed to nucleation of soluble Cu­(I) into Cu_2_O.

**Figure 4:**
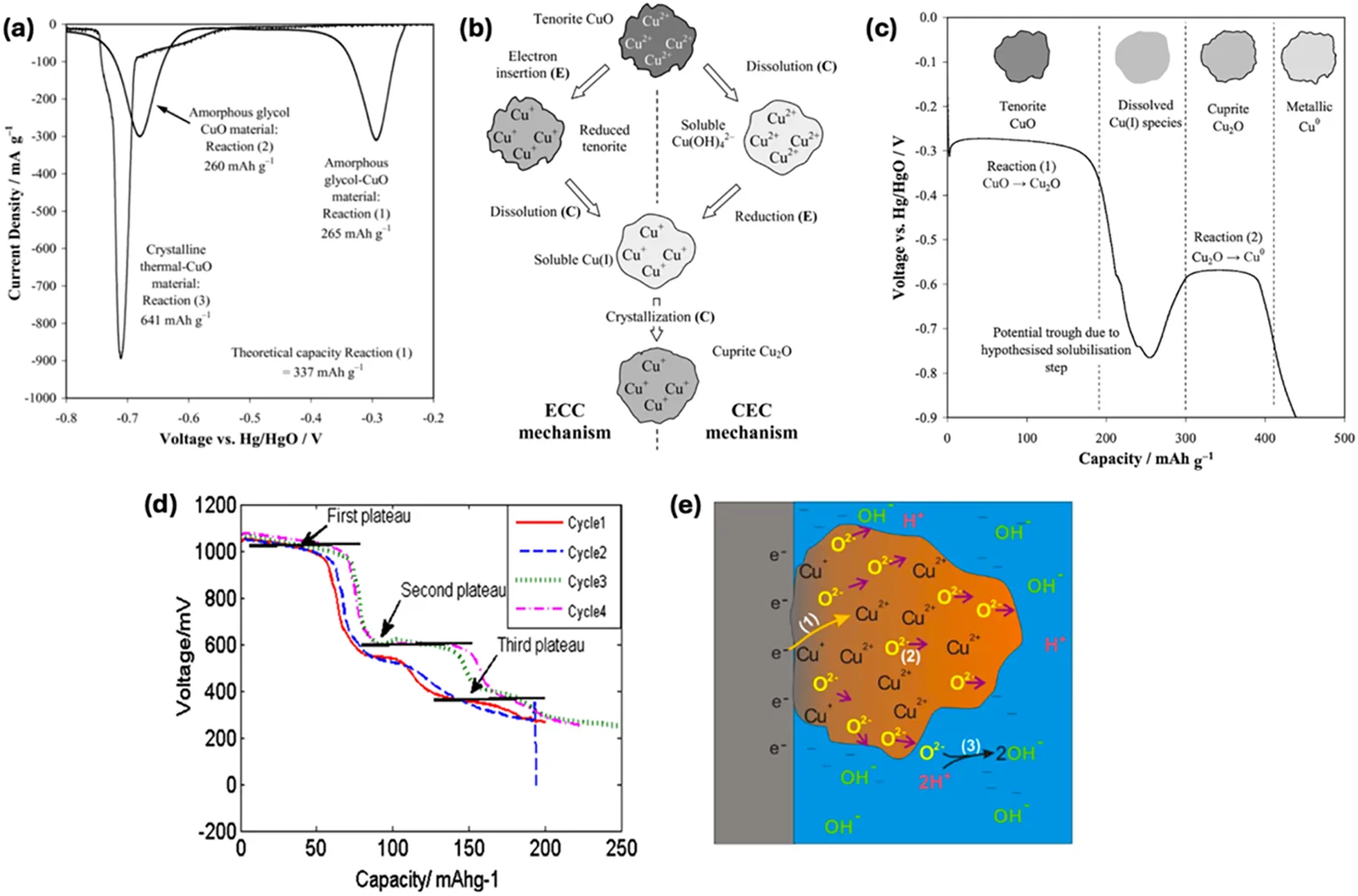
Studies of reduction of CuO in alkaline electrolyte. (a) Linear sweep voltammetry (LSV) of crystalline and amorphous CuO in 9 M KOH solution.^30^ (b) Proposed ECC dissolution–precipitation mechanism for reaction ([Disp-formula eq6]).^30^ (c) Mechanism of the discharge voltage profile.^30^ (a–c) Reproduced from ref 30 with permission. © The Electrochemical Society. Reproduced by permission of IOP Publishing Ltd. All rights reserved. (d) Voltage profile during the discharge of CuO nanorods cycled against Zn. [Reprinted by permission from Springer Nature: Journal of Solid State Electrochemistry, “Synthesis and elucidation of electrochemical characteristics of nanorods, microsized and nanosized CuO as cathode materials for Zn/CuO alkaline battery”, Zeraatkish et al., vol. 19, pp. 2155–2165, Copyright (2015)][Bibr ref27] (e) Proposed mechanism of discharge in high pH electrolytes, with schematic showing low O^2–^ ion concentration gradient inside the particle. [Reproduced from ref 31 with permission. © The Electrochemical Society. Reproduced by permission of IOP Publishing Ltd. All rights reserved.][Bibr ref31]

Zeraatkish et al.^27^ investigated CuO cathodes in 4 M NaOH across different particle morphologies, observing voltage features consistent with the ECC mechanism plus a third discharge plateau. Nanorod CuO exhibited higher capacity than nanoparticles and microsized particles (Figure 4d). While the first discharge of all morphologies showed a single voltage plateau, later cycles exhibited three distinct plateaus; the upper plateau was attributed to Cu_2_O_3_ reduction, though this was not confirmed by characterization.

Wang et al.^31^ confirmed the two-step CuO reduction using X-ray diffraction (XRD) and electrolysis experiments and proposed a three-stage solid-state mechanism: (i) electrons reduce Cu^2+^ to Cu^+^ at the current collector; (ii) Cu–O bonds break, allowing O^2–^ to diffuse from the CuO crystal toward the particle-electrolyte interface; (iii) O^2–^ reacts with H^+^ to form H_2_O. This provided atomistic insights into oxygen-proton interaction, complementing the ECC mechanism.

Wang et al.^31^ also reported the influence of scan rate, particle size, pH, and anions on cathodic peak shape. At high pH (6 M KOH, 1 M LiOH), low H^+^ concentration limits the O^2–^-H^+^ surface reaction rate, reducing the O^2–^ gradient (Figure 4e) and requiring higher overpotentials, producing two distinct CV peaks. High scan rates (100 mV/s) and large particle size have the same effect. At low pH (0.1 M KCl), high H^+^ concentration accelerates the surface reaction, steepening the O^2–^ gradient and lowering overpotential, producing merged peaks. Low scan rates (1 mV/s) and small particle size similarly promote merging. Cl^–^ ions enhanced H^+^ accumulation at the particle surface, further promoting CuO reduction. Na_2_S addition also promoted reduction, attributed to formation of conductive Cu_2_S that lowered interparticle contact resistance.

#### 2.2.3 Solubility of Cu in Alkaline Electrolyte

Understanding dissolved species is crucial as they directly influence cycling efficiency and cycle life, forming an important part of the electrochemical mechanism but also risking irreversible capacity loss if they migrate to electrochemically inaccessible locations.

Miller (1969)^25^ conducted an early RRDE study in 1N NaOH, sweeping the copper disk from −0.6 V to +0.6 V vs saturated calomel electrode (SCE) at 1000 rpm with the gold ring held at fixed potentials of 0 V, + 0.55 V, and −0.9 V vs SCE (Figure 5a). During the first anodic transition, 79% of Cu oxidized to Cu_2_O (passivating film) while 21% dissolved as soluble Cu­(I), indicated by peak a at 0 V. Further anodization oxidized Cu­(I) to Cu­(II) in an active dissolution stage, with ring collection efficiency reaching the theoretical maximum (33%) and no significant film formation, confirmed by peak f at −0.9 V corresponding to reduction of soluble Cu­(II) to Cu metal. Further anodization produced unstable Cu­(III), whose dissolution was confirmed by peak b corresponding to Cu­(III)→Cu­(II) reduction at the ring. Miller concluded that Cu­(I) and Cu­(III) undergo partial dissolution due to surface passivation, Cu­(II) undergoes active dissolution, and Cu­(III) is short-lived (lifetime ∼10^–2^ s). Ambrose et al. (1973)^22^ proposed that the soluble Cu­(III) species is [Cu­(OH)_4_]^−^. Soluble Cu­(II) formation during Cu oxidation was further confirmed by RRDE in Schorr et al.^11^ and Arnot et al.,^16^ though RRDE studies of soluble species during CuO reduction remain unexplored.

**Figure 5:**
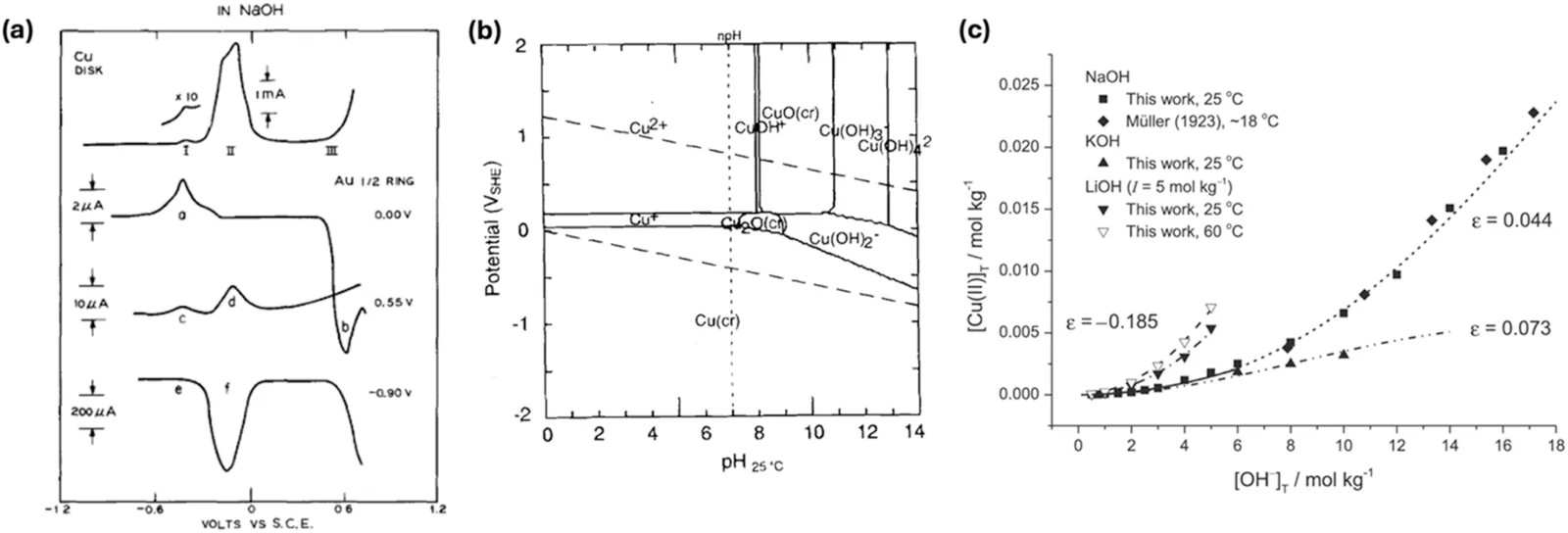
Nature of dissolved Cu species in solution. (a) RRDE experimental results for copper disk in 1N NaOH. Reproduced from ref 25 with permission. © The Electrochemical Society. Reproduced by permission of IOP Publishing Ltd. All rights reserved.^25^ (b) Pourbaix diagram of copper at 25 °C with [Cu­(aq)]_total_ = 10^–8^ mol/kg. Reproduced from ref 34 with permission. © The Electrochemical Society. Reproduced by permission of IOP Publishing Ltd. All rights reserved.^34^ (c) Solubility of CuO in alkaline solutions at different concentrations and temperatures. [Reprinted from Hydrometallurgy, vol. 147, M. Navarro, P. M. May, G. Hefter, and E. Königsberger, “Solubility of CuO(s) in highly alkaline solutions”, pp. 68–72, Copyright (2014), with permission from Elsevier.][Bibr ref35]

Beverskog and Puigdomenech (1997)^34^ revised the Cu Pourbaix diagram using modern thermodynamic data and cation hydrolysis theory (Figure 5b), for the first time accounting for dissolved [Cu­(OH)_2_]^−^ and four soluble Cu­(II) species: [Cu­(OH)_3_]^−^, [Cu­(OH)_4_]^2–^, [Cu_2_(OH)_2_]^2+^, and [Cu_3_(OH)_4_]^2+^. Including [Cu­(OH)_2_]^−^ altered the Cu_2_O stability region: at pH > 13, increasing potential produces [Cu­(OH)_2_]^−^ then [Cu­(OH)_4_]^2–^, bypassing crystalline CuO formation. They also concluded that crystalline Cu­(OH)_2_ is thermodynamically unstable relative to CuO and excluded it from the revised diagram.

Navarro et al. (2014)^35^ measured CuO solubility in NaOH, LiOH, and KOH at 25 and 60 °C (Figure 5c), finding [Cu­(OH)_4_]^2–^ as the dominant dissolved Cu­(II) species under all conditions, with solubility increasing with OH^–^ concentration and temperature following LiOH ≫ NaOH > KOH. UV–vis spectra of saturated solutions confirmed this with a consistent absorbance maximum at 638 nm. The higher LiOH solubility was attributed to stronger Li^+^-[Cu­(OH)_4_]^2–^ ion pairing, quantified by Specific Ion Interaction Theory (SIT).

In summary, Cu(0) oxidation (charging) in alkaline solution involves duplex film formation and transient Cu­(III) species, while CuO reduction (discharge) involves solid-state and dissolution–precipitation mechanisms (ECC) with Cu­(I) intermediates and resistive Cu_2_O. While CuO discharge has been studied in the context of battery performance, Cu charging is best summarized by the works of He et al. and Miller et al., both of which focus on flat Cu metal substrates. The He work deeply characterized the duplex film, mostly leaving aside dissolved species. However, RRDE work clearly indicates Cu­(I), Cu­(II), and Cu­(III) species in solution. Understanding these complex mechanisms, particularly charging as summarized in Figure 1, requires further work in the context of batteries.

### 2.3 Recent Advancements

Zhu et al. (2019)^36^ reported reversible Cu cycling in 1 M KOH but not in 1 M ZnSO_4_, as confirmed by in situ Raman spectroscopy and ex situ XRD. The Zn–Cu battery in 1 M KOH delivered ∼300 mAh/g over 200 cycles, whereas capacity dropped to zero after only 10 cycles in 1 M ZnSO_4_ (Figure 6a). Reversibility in alkaline electrolyte was attributed to suppression of Cu^2+^ dissolution. By solubility product theory, soluble Cu^2+^ concentration in 1 M KOH is 10^–14^ times that in neutral solution.

**Figure 6:**
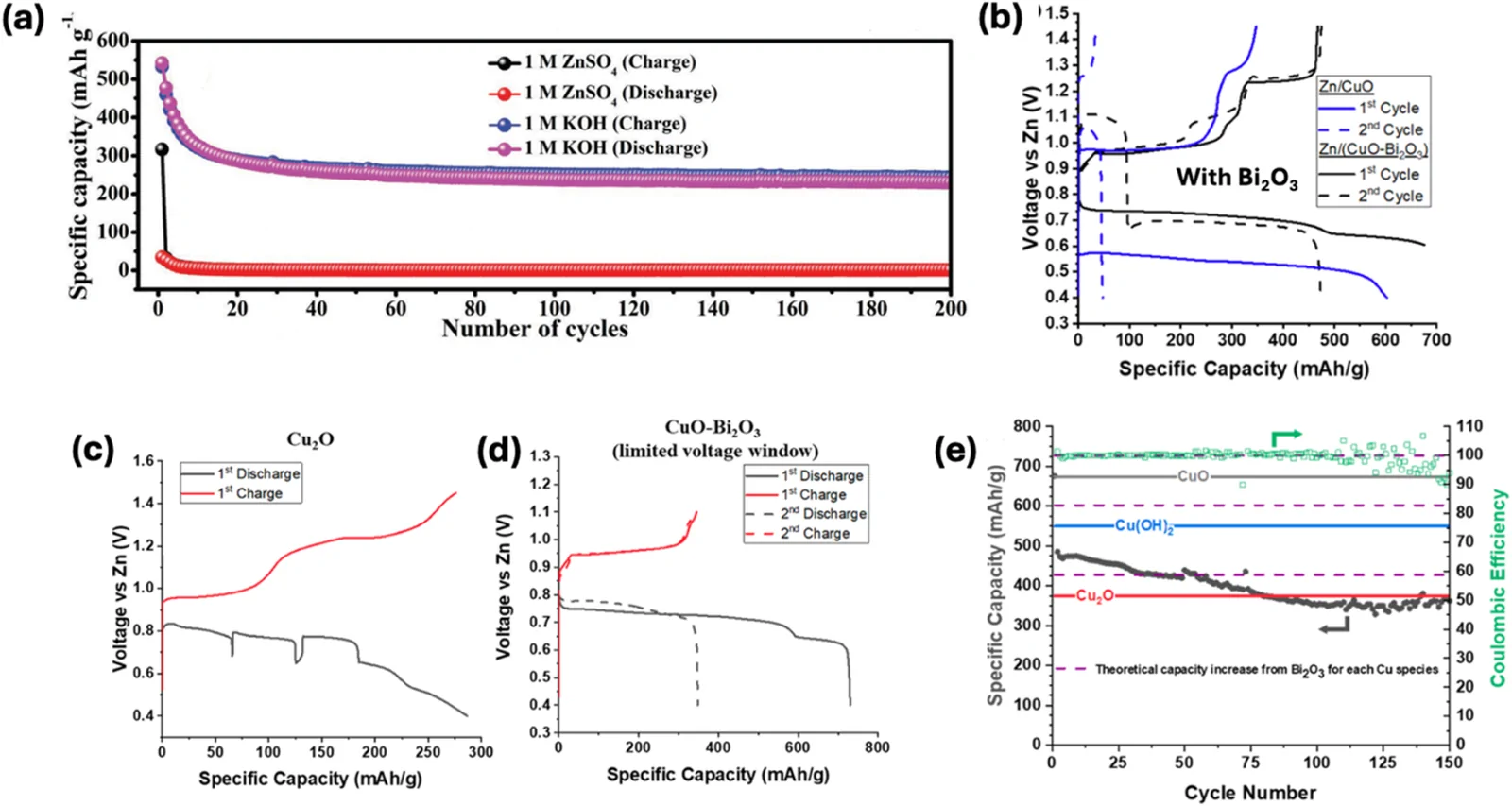
Electrochemical performance of Cu-based cathodes in secondary batteries. (a) Specific capacity versus cycle number for the Cu–Zn battery with 1 M ZnSO_4_ and 1 M KOH electrolytes at a current rate of 1A/g. Reproduced with permission from Zhu et al., Advanced Functional Materials, 29, 1905979 (2019). © 2019 Wiley-VCH Verlag GmbH & Co. KGaA, Weinheim.^36^ (b) Voltage profiles of a Zn-CuO cell with and without Bi_2_O_3_ additive.^11^ Voltage profile versus specific capacity of cathodes composed of (c) Cu_2_O without additive, exhibiting voltage dips,^11^ and (d) CuO with Bi_2_O_3_ additive, showing no voltage dips.^11^ (e) Specific capacity and coulombic efficiency versus cycle number for Zn/(CuO-Bi_2_O_3_) cell.^11^ (b–e) Reprinted with permission from Schorr et al., ACS Applied Energy Materials, 4, 7073–7082 (2021). Copyright 2021 American Chemical Society.

Schorr et al.^11^ incorporated 10 wt % Bi_2_O_3_ into CuO cathodes based on prior work with MnO_2_ cathodes.^10,37^ With the Bi additive, CuO cathodes cycled reversibly in alkaline Zn batteries (Figure 6b), whereas cathodes without additive failed rapidly. Beyond enabling rechargeability, Bi_2_O_3_ suppressed voltage dips during discharge (Figure 6c,d). Capacity fade persisted at 100% cathode DOD (Figure 6e), but cycling at 30% DOD was stable for 250 cycles at 140 Wh/L, despite low Zn anode DOD (∼10%). A high cathode areal capacity of 40 mAh/cm^2^ was also demonstrated.

Based on in situ energy-dispersive X-ray diffraction (EDXRD) measurements, Schorr et al.^11^ reported that CuO was not the final oxidation product, suggesting instead Cu­(OH)_2_, which was amorphous and undetectable by EDXRD. Wygant et al.^39^ supported this interpretation, attributing ex situ XRD detection of CuO to dehydration of Cu­(OH)_2_ during post-mortem analysis. Direct in situ characterization of Cu­(OH)_2_ formation during charging would resolve the ongoing debate about the charge product. Schorr et al.^11^ suggested that Bi_2_O_3_ enabled rechargeability by decreasing cathode charge transfer resistance and reducing soluble copper species formation, likely through complexation of Bi­(III) with the copper surface (Figure 7a). It should be noted that, when present, Bi_2_O_3_ is also electrochemically active and reduces to Bi(0) at the end of discharge, resulting in some additional capacity at lower voltages. During subsequent charging, the resulting Bi metal is oxidized to Bi_2_O_3_. Wygant et al.^39^ found that good cycling stability was achieved with as little as 1 wt % Bi_2_O_3_, particularly when the electrolyte was presaturated with Bi_2_O_3_. They proposed that soluble Bi^3+^ during discharge improves Cu^2+^→Cu^1+^ conversion, while during charge it reduces Cu^2+^ solubility and stabilizes the Bi–Cu­(OH)_2_ product to inhibit CuO formation. The soluble form of Bi^3+^ in alkaline electrolyte is the tetrahydroxidobismuthate­(III) anion [Bi­(OH)_4_]^−^, sometimes written in dehydrated form as BiO_2_^–^. Loss of Bi^3+^ by diffusion from the electrode and pore space and into the bulk electrolyte, separator, and anode compartment was identified as a potential contributor to cell failure, motivating exploration of polymer gel electrolytes to suppress Bi^3+^ migration, with promising results.

**Figure 7:**
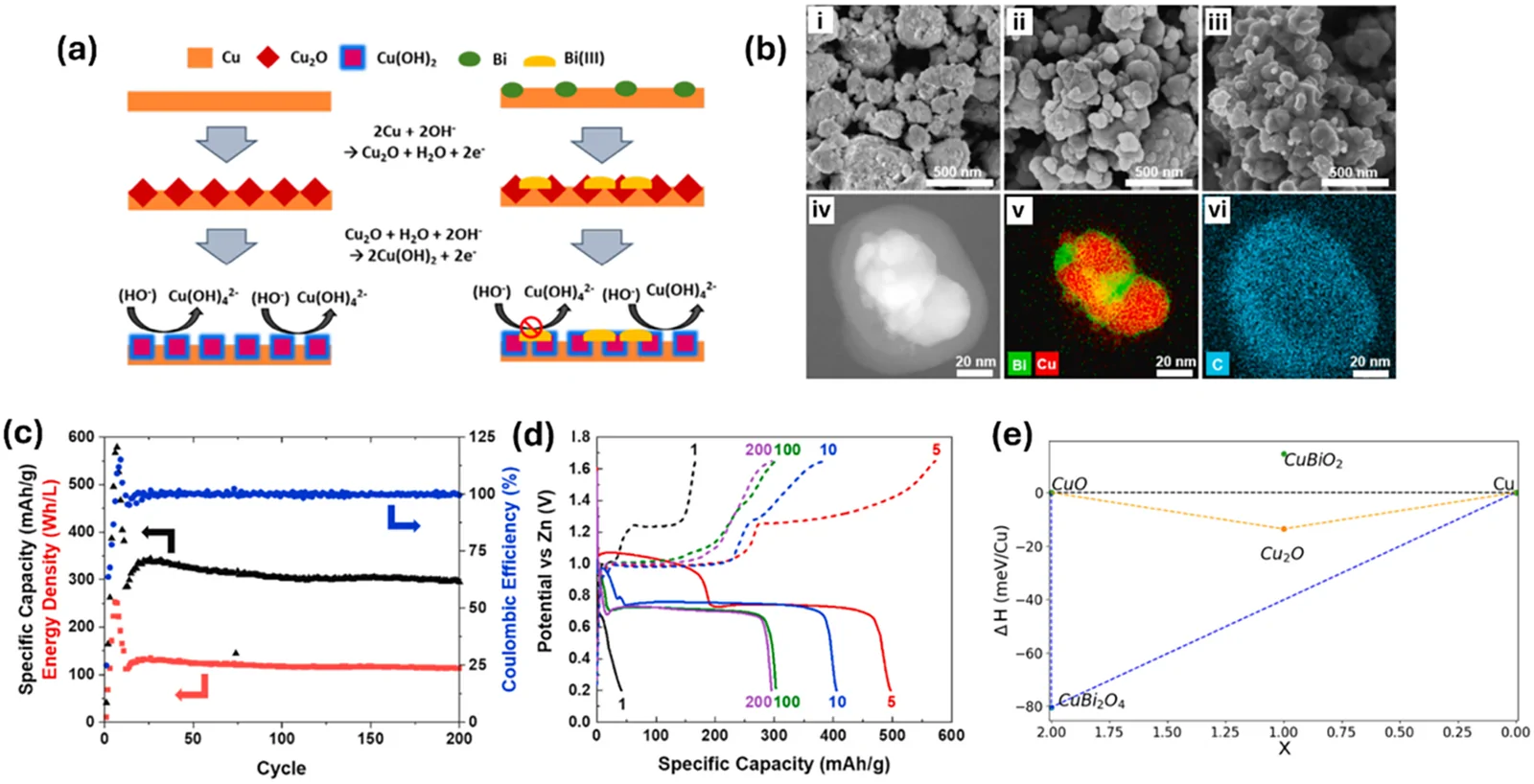
Effects of additives on CuO electrochemical performance. (a) Schematic representation of the oxidation process of CuO with and without Bi_2_O_3_ additive. Reprinted with permission from Schorr et al., ACS Applied Energy Materials, 4, 7073–7082 (2021). Copyright 2021 American Chemical Society.^11^ (b) Scanning electron microscopy (SEM) and transmission electron microscopy (TEM) analysis of the CuO/Bi_2_O_3_ cathode material: (i) SEM image of the as-prepared CuO/Bi_2_O_3_ composite, (ii) SEM image of CuO/Bi_2_O_3_ coated with polydopamine. (iii) SEM image of Cu/Bi coated with carbon after heat treatment. (iv) TEM image of a carbon-coated Cu/Bi particle. (v) Energy-dispersive X-ray spectroscopy (EDS) map showing the distribution of Bi and Cu. (vi) EDS map confirming the carbon shell encapsulating the Cu/Bi particle.^16^ (c) Cycling results for a carbon-coated CuO/Bi_2_O_3_–Zn battery.^16^ (d) Voltage profile for the carbon-coated CuO/Bi_2_O_3_–Zn battery at different cycle numbers.^16^ (b–d) Reprinted from Journal of Power Sources, vol. 529, Arnot et al., “Rechargeable alkaline Zn–Cu batteries enabled by carbon coated Cu/Bi particles”, p. 231168, Copyright (2022), with permission from Elsevier. (e) Formation enthalpies of Cu_2_O, CuBiO_2_ and CuBi_2_O_4_ per Cu atom. X is the oxidation state of Cu. Reprinted with permission from ref 38. Copyright 2025 The Electrochemical Society. Reproduced by permission of IOP Publishing Ltd. All rights reserved.^38^

Further addressing solubility, Arnot et al.^16^ prepared metallic Cu/Bi particles with carbon to suppress soluble cuprate and bismuthate migration while maintaining hydroxide transport (Figure 7b). Carbon coating improved cycling stability and inhibited Cu_2_O accumulation, but active materials solubilized even through 20 nm coatings, partly due to volumetric expansion upon conversion to oxide phases. Their results suggest that in situ prepared carbon-coated CuO can cycle without Bi additive, contrasting with Schorr et al.^11^ Arnot et al.^16^ assembled the carbon-coated cathodes in the discharged state. At 0.1C rate, specific capacity reached 550 mAh/g by cycle 7, then stabilized at 300 mAh/g with 89% retention from cycles 20–200 at C/5 (Figure 7c). Two discharge plateaus were observed at cycle 5: an upper plateau at ∼1 V attributed to Cu­(OH)_2_ reduction and a lower plateau at ∼0.8 V attributed to CuO and Cu_2_O reduction (Figure 7d). The upper plateau contribution diminished after cycle 10. Ex situ XRD showed CuO as the dominant Cu­(II) species in later cycles, with metallic copper at end of charge attributed to material isolation, and a minor CuBi_2_O_4_ phase in charged-state electrodes.

Acharya et al.^38^ applied density functional theory (DFT) to investigate discharge mechanisms in alkaline CuO cathodes with carbon and Bi_2_O_3_ additives, finding that stable mixed Cu–Bi species such as CuBi_2_O_4_ follow an alternative redox pathway that suppresses resistive Cu_2_O accumulation (Figure 7e). Wygant et al.^39^ demonstrated that decreasing Bi_2_O_3_ loading to as little as 1 wt % in a Bi-saturated electrolyte improved volumetric energy density and cycle retention relative to conventional 10 wt % loading.

Duay et al.^40^ developed rechargeable Cu–S solid-state cathodes for alkaline batteries, identifying Cu_2_S as an effective active material (Figure 8a) that delivered ∼150 mAh/g at 50% cathode DOD over 450 cycles. At 100% DOD, cathodes failed due to a gradual shift from S to Cu redox chemistry, caused by sulfur disproportionation into irreversible sulfite species that released bound Cu ions. Meng et al. (2020)^41^ evaluated CuO nanorods in mildly acidic ZnSO_4_ for aqueous zinc-ion batteries, reporting that after the first discharge, redox proceeded reversibly only between Cu_2_O and Cu, delivering 219 mAh/g at 0.3 A/g, supported by CV showing one anodic and one cathodic peak (Figure 8b).

**Figure 8:**
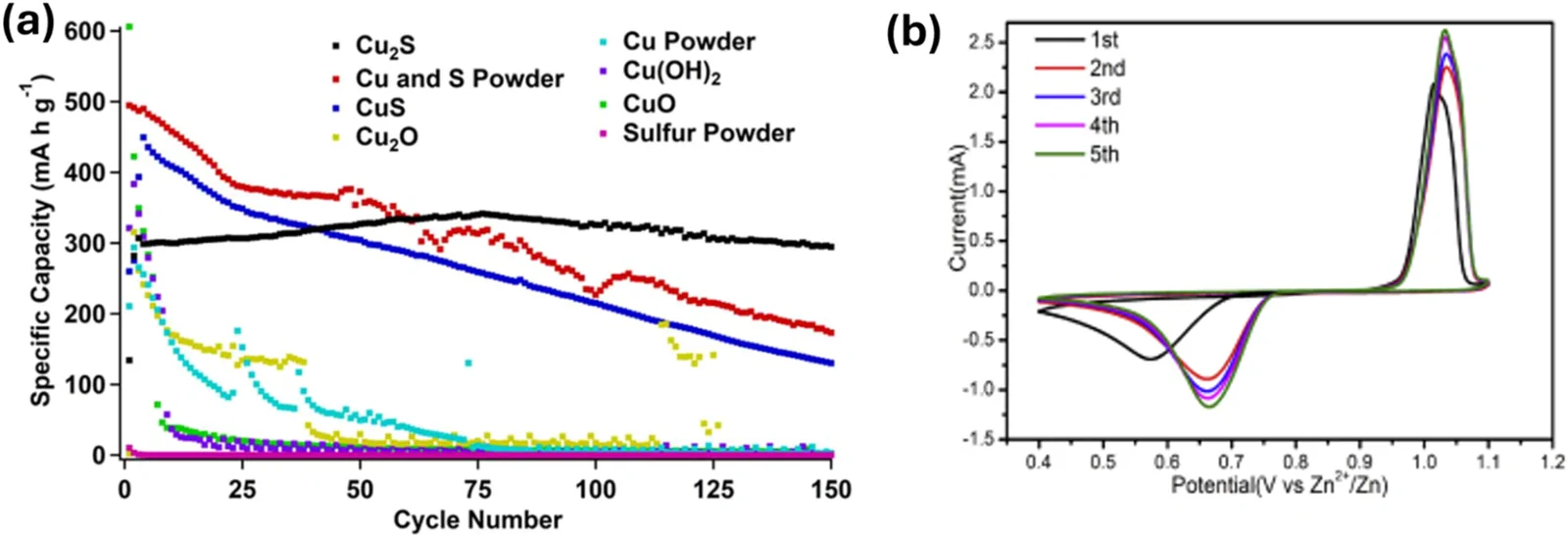
Electrochemical characterization of copper-based electrodes and their performance in nonalkaline electrolytes. (a) Specific capacity versus cycle number for various Cu and S materials used as the cathode active material against a Zn anode. Reprinted from Duay et al., *Journal of the Electrochemical Society*, 166, A687 (2019). © The Author(s) 2019. Published by ECS. Licensed under CC BY-NC-ND 4.0.^40^ (b) CV profile of a CuO electrode at 0.1 mV/s in 3 M ZnSO_4_ solution. Reprinted with permission from Meng et al., *Materials Today Energy*, 15, 100370 (2020). Copyright 2020 Elsevier.^41^

Among recent Cu-based cathodes for rechargeable batteries, CuO with Bi_2_O_3_ additives is most promising due to its high energy density and areal capacity when the electrochemical environment is carefully engineered. However, shallow DOD, unclear high-DOD failure mechanisms, and resistive Cu_2_O accumulation remain significant barriers to commercial development, as outlined among the challenges in Figure 1.

## 3 Economic and Energy Density Considerations

We performed a material-cost-based economic analysis to evaluate the Zn-CuO battery system and propose practical strategies toward commercialization, summarized in Figure 9. Schorr et al. demonstrated CuO cathode cycling at 40 mAh/cm^2^ and reported an energy density of 260 Wh/L at 10% Zn anode DOD.^11^ In their study of intercalation-type Zn-MnO_2_ batteries, Shang et al. suggested that energy density levels off near a cathode areal capacity of 3 mAh/cm^2^, making higher values unnecessary.^42^ We similarly analyzed the influence of cathode areal capacity and anode DOD on volumetric energy density, gravimetric energy density, and material cost per capacity for conversion-type Zn-CuO batteries. Full details of the cell-level calculations, including input parameters, material properties, and cost assumptions, are provided in Supporting Information: Table S1 and eq S1–S22. This analysis uses Zn utilization (Zn DOD) and cathode areal capacity (which is a metric including both cathode loading and cathode thickness) as the primary variables affecting cell design. The CuO cathode was assumed to cycle at theoretical capacity (674 mAh/g) because this is the stated goal of recent literature. High cycle life is desired, and will determine the levelized cost of storage, based on the energy storage cost in USD/kWh. However, different grid storage applications will have different cycle life requirements, and thus we ultimately use energy storage cost as the model output. The effect of electrolyte volume is a current area of research.

**Figure 9:**
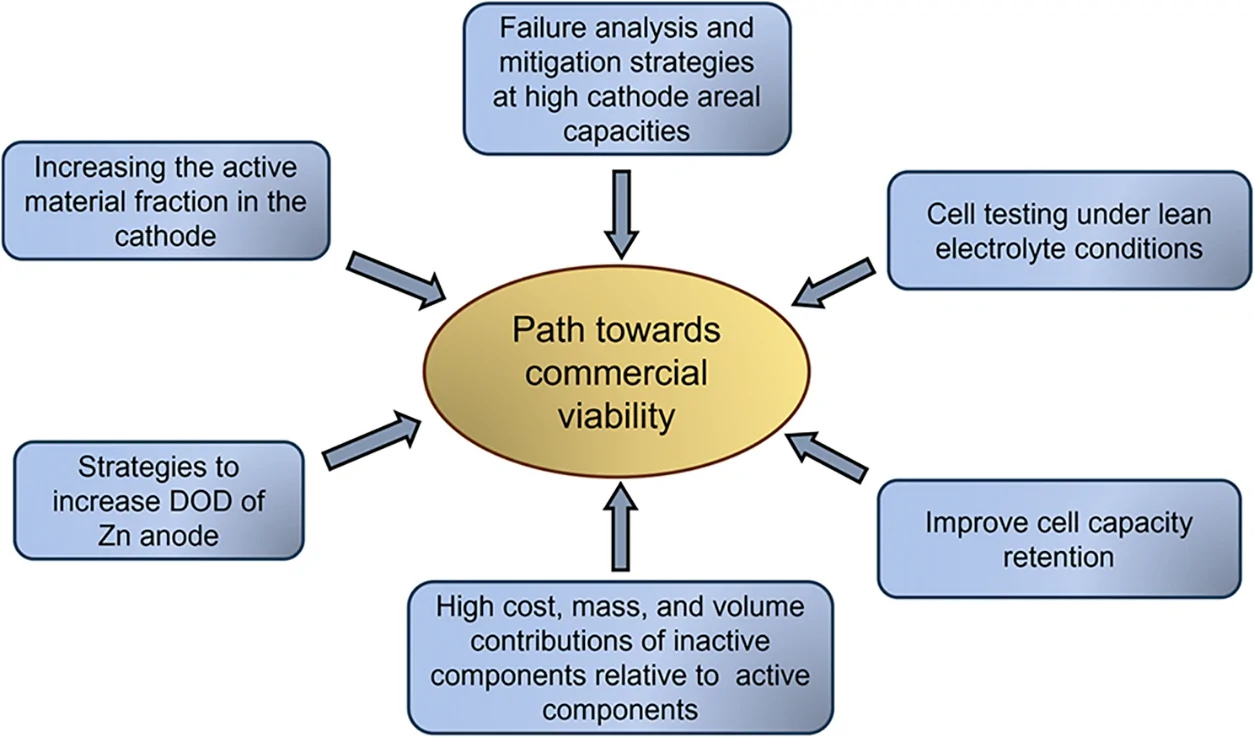
Promising directions toward commercial viability of rechargeable alkaline Zn-CuO system.

For battery design, one desires to achieve the greatest fraction of mass and volume to be occupied by active materials, minimizing inactive components to the extent that performance is not reduced. At 15% Zn DOD, gravimetric energy density increases rapidly with cathode areal capacity approximately up to 20 mAh/cm^2^ (Figure 10a), where inactive components (separators, current collectors) dominated cell mass (Figure 10b). Beyond 20 mAh/cm^2^, active material contributions outweighed inactive components, yielding more gradual improvement. A similar trend held for volumetric energy density (Figure 10c): at low areal capacities, inactive component volume dominated (Figure 10d), but a sharp increase was observed up to about 25 mAh/cm^2^, followed by gradual improvement beyond that point.

**Figure 10:**
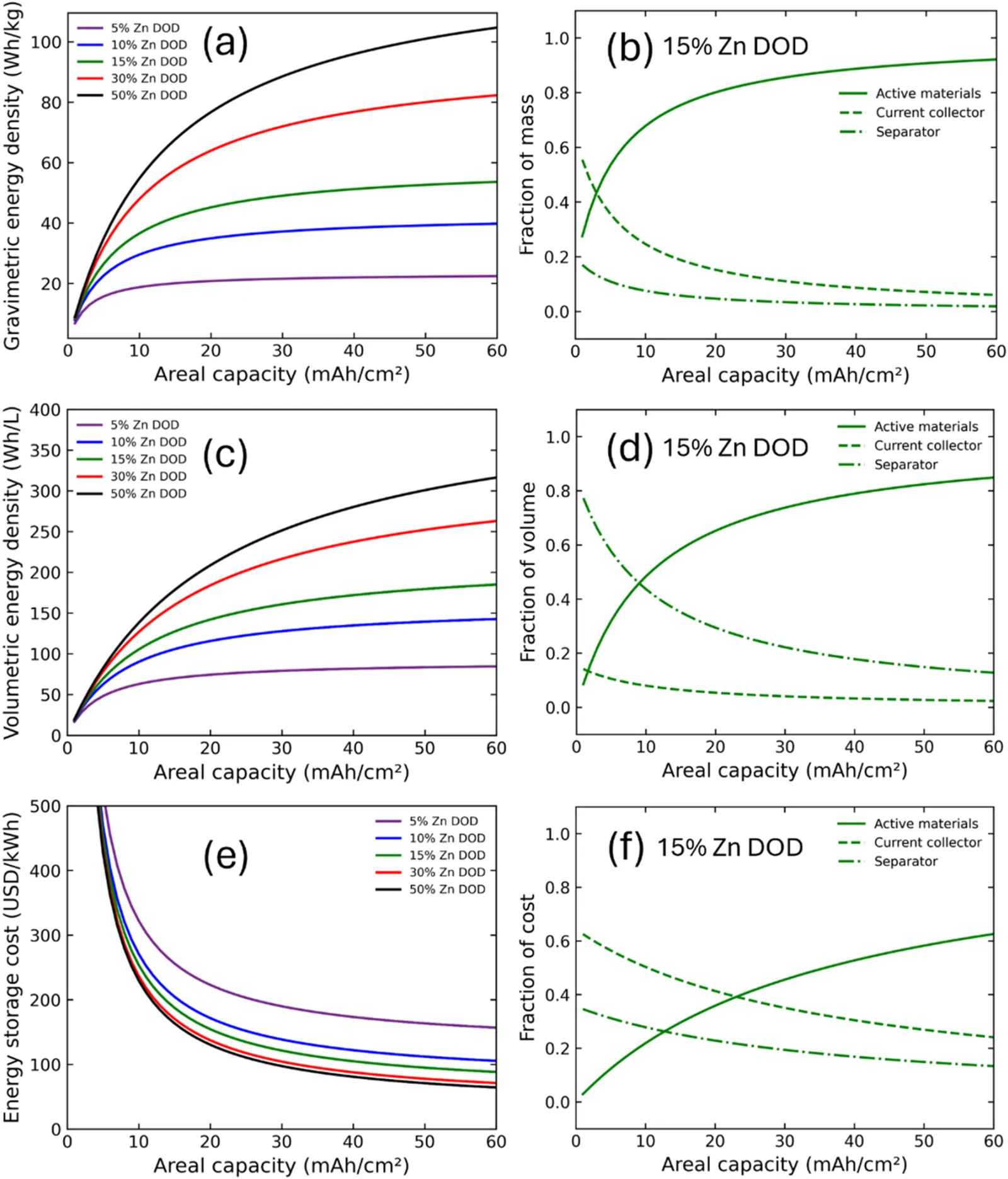
Energy density, cost, and component fractions as a function of CuO cathode areal capacity and Zn anode DOD. (a) Gravimetric energy density. (b) Mass fraction of cell components (at Zn DOD of 15%). (c) Volumetric energy density. (d) Volume fraction of cell components (at Zn DOD of 15%). (e) Energy storage cost. (f) Cost fraction of cell components (at Zn DOD of 15%).

Below 10 mAh/cm^2^, inactive component costs drove energy storage costs above 200 USD/kWh for all Zn DODs, with costs becoming astronomical at lower areal capacity. Increasing cathode areal capacity reduced costs significantly (Figure 10e), and areal capacities exceeding 40 mAh/cm^2^ were projected to achieve below 100 USD/kWh even at a conservative 15% Zn DOD (Figure 10f), as active material contributions to delivered capacity became dominant relative to the fixed inactive component inventory. Increasing Zn DOD to 50% yielded further dramatic improvements: 316 Wh/L volumetric energy density, 105 Wh/kg gravimetric energy density, and 64 USD/kWh storage cost. However, high Zn utilization is constrained by shape change, passivation, and dendrite formation, and will require advancements in Zn anodes as well.^43^

The projected storage costs are sensitive to active material pricing, and to assess the level of sensitivity we varied them in the model at the condition of 40 mAh/cm^2^ CuO cathode areal capacity and zinc DOD of 15%. A 25% increase in CuO and Bi_2_O_3_ prices increased the projected storage cost modestly from 104.8 to 109 USD/kWh and 105.41 USD/kWh, respectively. As expected, cost was more sensitive to CuO price as this material is a far larger fraction of the cathode formulation. In the model, a Bi_2_O_3_ weight fraction of 0.1 was used, as this was the value used by Schorr et al.^11^ However, later references have used amounts of Bi_2_O_3_ down to 1 wt %, and sensitivity to Bi_2_O_3_ price declines with the amount used.^39^ Because the fundamental mechanism of the Bi_2_O_3_ effect is not fully described, the threshold value required remains undefined. This analysis confirmed that neither CuO nor Bi_2_O_3_ pricing is the dominant cost driver at a cathode areal capacity of 40 mAh/cm^2^ and zinc DOD of 15%.

Under lean electrolyte conditions suitable for practical systems, the minimum theoretical electrolyte volume required at 15% Zn DOD is approximately 1.7 mL/Ah (assuming 35% KOH, 30% separator porosity, and 40 mAh/cm^2^ cathode areal capacity). This represents the pore-filling volume only, but commercial cells require additional electrolyte for better ionic transport and electrode wetting. This theoretical minimum is far below the excess used by Schorr et al.^11^ (65 μL/mAh, 45 wt % KOH and 40 mAh/cm^2^ cathode areal capacity). Optimizing toward lean conditions would further improve energy density, though a minimum electrolyte volume may be required to sustain CuO redox reactions.

## 4 Future Outlook

Recent progress has demonstrated CuO cathode rechargeability with Bi_2_O_3_ additives and carbon coatings. Future efforts should focus on in situ characterization to directly observe Cu­(OH)_2_ formation during charge and clarify the role of Bi_2_O_3_ in stabilizing specific Cu phases, as resolving the charge product identity is a prerequisite for rational electrode engineering. To more fully describe the complicated Cu/CuO electrochemistry in alkaline electrolyte, operando Raman spectroscopy can identify surface-bound intermediates and track phase evolution during cycling, while X-ray absorption near-edge structure (XANES) and extended X-ray absorption fine structure (EXAFS) can track Cu oxidation state evolution with respect to voltage with high chemical sensitivity. RRDE studies should be employed to confirm the soluble Cu­(I) intermediate species, which subsequently crystallize to form solid Cu_2_O through the electrochemical-chemical-crystallization (ECC) mechanism, attributed to the characteristic voltage dip. RRDE measurements are well suited to investigate whether Bi_2_O_3_ suppresses the voltage dip by providing heterogeneous nucleation sites for Cu_2_O crystallization. The electrochemical mechanisms of the Cu­(I) intermediates have importance for achieving long-term rechargeability with good capacity retention.

For battery development, post-mortem analysis of electrodes cycled at high DOD will be critical to identify failure mechanisms including active material isolation, irreversible phase formation, and structural degradation. SEM coupled with EDS and XRD is recommended to characterize morphological evolution and phase distribution in cycled electrodes. High-resolution transmission electron microscopy (HRTEM) coupled with selected-area electron diffraction (SAED) can be employed to resolve the duplex film structure at the nanoscale within individual particles as a function of DOD. Suppressing soluble Cu and Bi species migration remains equally important and advanced separators or polymer gel electrolytes that permit hydroxide transport while limiting soluble species diffusion into the bulk electrolyte and anode compartment are a promising direction warranting further development. Achieving economic viability will require translating these mechanistic advances into stable cycling at high cathode DOD and cathode areal capacities exceeding 40 mAh/cm^2^, which are the conditions under which the cost and energy density targets identified in this review become accessible.

## 5 Conclusion

Cupric oxide cathodes with Bi_2_O_3_ additives represent the most promising path toward rechargeable alkaline Zn-CuO batteries for stationary energy storage, combining a favorable material cost basis, inherent safety from aqueous electrolytes, and high theoretical capacity. The electrochemical mechanisms of Cu and CuO in alkaline electrolyte were reviewed, revealing a multistep oxidation pathway involving duplex film formation and transient dissolved species, and a two-step discharge pathway proceeding through the electrochemical-chemical-crystallization (ECC) mechanism with soluble Cu­(I) intermediates and resistive Cu_2_O accumulation. The unresolved nature of the charge product, CuO or Cu­(OH)_2_, has implications for understanding capacity fade and designing effective Bi_2_O_3_ modification strategies. Poorly controlled dissolution and migration of soluble Cu and Bi species out of the electrode and into the bulk electrolyte, separator, and anode compartment is a likely mechanistic contributor to capacity fade, and suppressing this migration is a prerequisite for stable cycling at high cathode DOD. The energy density and cost model analysis demonstrated that cathode areal capacity and Zn anode utilization are the dominant variables governing both energy density and storage cost, with areal capacities exceeding 40 mAh/cm^2^ required to achieve costs below 100 USD/kWh. Future research should focus on identifying and mitigating degradation mechanisms at high cathode utilization, developing approaches to suppress soluble species migration, and advancing Zn anode performance. These are advances that together can make the favorable material cost basis of the Zn-CuO system accessible at the system level.

## Supplementary Material



## Notes

Cell-level energy density and cost model: basis and assumptions; physical properties, compositions, and costs of cell components (Table S1); symbol definitions; equations for gravimetric energy density, volumetric energy density, cathode areal loading, cathode thickness, and energy storage cost (Equations S1–S22) (PDF)
