# Supplementary material for: From Mechanisms to Commercialization: CuO Positive Electrodes for Rechargeable Alkaline Zinc Batteries
Source: ACS Omega. 2026 Aug 27;11(35):52257–68. doi: 10.1021/acsomega.6c04389 (PMC13563622; doi:10.1021/acsomega.6c04389)
Supplement: Supplementary file 1 [file ao6c04389_si_001.pdf]

Supplemental Information

From Mechanisms to Commercialization: CuO Positive Electrodes for Rechargeable Alkaline Zinc Batteries

Yogeshwaran Agilan [1], Bryan R. Wygant [2], Timothy N. Lambert [3,4*], and Joshua W. Gallaway [1,*]

1. Department of Chemical Engineering, Northeastern University, 360 Huntington Avenue, Boston, MA 02115, USA.

2. Nanoscale Sciences Department, Sandia National Laboratories, Albuquerque, New Mexico 87185, United States of America.

3. Department of Photovoltaics and Materials Technology, Sandia National Laboratories, PO Box 5800, Albuquerque, NM 87185, USA.

4. Center for Integrated Nanotechnologies, Sandia National Laboratories, PO Box 5800, Albuquerque, NM 87185.

Corresponding Authors
* Email: j.gallaway@northeastern.edu, tnlambe@sandia.gov

Basis and Assumptions

The cost analysis was performed based on the cost of materials alone including active materials, binder, additives, surfactant, electrolyte, separators, and current collectors. Cell housing mass and cost was not accounted for both gravimetric energy density and cost calculations. A superficial electrode area of 6 in x 3 in (15.24 cm x 7.62 cm) was selected as the unit cell basis. The cell configuration including electrode formulations and separator arrangements, followed the design demonstrated by Schorr et al.[S1] For all the calculations, the cathode depth of discharge (DOD) of 100% was considered, corresponding to the two-electron CuO reaction. A nominal voltage of 0.9 V was used, corresponding to the average discharge potential 1.05 V vs Zn for the first electron capacity and 0.73 V for the second electron capacity.[S2] For volumetric energy density calculations, the electrolyte volume was limited to that required to fill the pore volume of the cathode, anode and separator layers, since the electrolyte primarily occupies the porous regions under starved electrolyte conditions.[S1] Material costs were obtained from bulk commodity databases and supplier platforms, based on the median pricing over the last one year period (March 2025-March 2026). Separator costs were estimated at 0.0001 USD/cm^2 of superficial area. The cost of water present in the polytetrafluoroethylene (PTFE, 60% dispersion in water) and in the 35 wt.% KOH electrolyte was excluded, as water represents a negligible contribution to the total material cost.

Table S1. Physical Properties, Compositions and Costs of Cell Components for Zn-CuO Alkaline Battery

<table>
<tr><td colspan="2">Cell nominal voltage (V)</td><td colspan="4">0.9</td></tr>
<tr><td colspan="2">Cathode depth of discharge (%)</td><td colspan="4">100</td></tr>
<tr><td colspan="2">Cathode porosity</td><td colspan="4">0.4</td></tr>
<tr><td colspan="2">Specific capacity of cathode active material</td><td colspan="4">674 mAh/g of CuO</td></tr>
<tr><td colspan="2">Anode porosity</td><td colspan="4">0.35</td></tr>
<tr><td colspan="2">Overall separator porosity</td><td colspan="4">0.3</td></tr>
<tr><td colspan="2">Specific capacity of anode active material</td><td colspan="4">820 mAh/g of Zn</td></tr>
<tr><td colspan="6">Chemicals – physical properties and cost</td></tr>
<tr><td>Component</td><td>Chemical name</td><td>Role</td><td>Weight fraction</td><td>Density (g/cm^3)</td><td>Cost (USD/g)</td></tr>
<tr><td rowspan="4">Cathode</td><td>Copper oxide (CuO)</td><td>Active material</td><td>0.55</td><td>6.31</td><td>0.00955[S3]</td></tr>
<tr><td>Graphite</td><td>Conductive additive</td><td>0.3</td><td>2.2136</td><td>0.000796[S4]</td></tr>
<tr><td>Bismuth oxide (Bi_2O_3)</td><td>Additive</td><td>0.1</td><td>8.93</td><td>0.007200[S5]</td></tr>
<tr><td>PTFE dispersion in water (60%)</td><td>Binder</td><td>0.05</td><td>1.83</td><td>0.01130[S6]</td></tr>
<tr><td rowspan="4">Anode</td><td>Zinc (Zn)</td><td>Active material</td><td>0.831</td><td>7.133</td><td>0.00275[S7]</td></tr>
<tr><td>Zinc oxide (ZnO)</td><td>Additive</td><td>0.098</td><td>5.6</td><td>0.00325[S8]</td></tr>
<tr><td>Sodium dodecylbenzenesulfonate (SDBS)</td><td>Surfactant</td><td>0.021</td><td>1.06</td><td>0.0018[S9]</td></tr>
<tr><td>Polytetrafluoroethylene (60 wt.% PTFE dispersion in water)</td><td>Binder</td><td>0.05</td><td>1.83</td><td>0.00618[S6]</td></tr>
</table>

Electrolyte	Aqueous potassium hydroxide (KOH)	Solute	0.35	1.33	0.000787[S10]

Separator - cost

Component	Name	Cost per superficial area (USD/cm^2)	Thickness (cm)	Mass per superficial area (g/cm^2)	Number of layers
Wicking agent	Celgard	0.0001	0.0075	0.002825	2
	Cellophane sheet	0.0001	0.0026	0.0039	2
	Kimwipe	0.0001	0.0075	0.00205	2

Current collector - cost

Component	Name	Cost (USD/g)	Thickness (cm)	Volume per superficial area (cm^3/cm^2)	Mass per superficial area (g/cm^2)
Anode current collector	Copper mesh	0.011[S11]	0.0130	0.00333	0.0276
Cathode current collector	Nickel mesh	0.015[S12]	0.0250	0.0031	0.0297
Tabs	Nickel tab	0.02 (USD/count)			

Symbols

A	Superficial area of the electrode pack (cm^2)
$C_{storage}$	Total cost per kWh (USD/kWh)
$C_{chemical}$	Total chemical cost (USD)
$C_{a,i}$	Cost of anode materials (USD/g)
$C_{c,i}$	Cost of cathode materials (USD/g)
$C_{current\ collector}$	Total cost of current collectors (USD)
C_e	Cost of electrolyte salt (USD/g)
$C_{mesh,i}$	Cost of current collector mesh (USD/g)
$C_{separator}$	Total separator cost (USD)
$C_{s,i}$	Cost of separators (USD/cm^2)
C_{tab}	Cost of a tab for current collectors (USD/count)
C_T	Total capacity of the battery (mAh)

$C_{unit\ cell}$	Total cost of a unit cell (USD)
D_a	Depth of discharge of the anode
D_c	Depth of discharge of the cathode
E	Nominal voltage
ED_g	Gravimetric energy density (Wh/kg)
ED_v	Volumetric energy density (Wh/L)
ε_a	Porosity of anode
ε_c	Porosity of cathode
ε_s	Net porosity of separator
$F_{a,i}$	Weight fraction of anode materials
$F_{c,i}$	Weight fraction of cathode materials
F_e	Weight fraction of electrolyte salt
L_c	Areal loading of the cathode (mAh/cm^2)
M_a	Mass of anode mixture (g)
M_c	Mass of cathode mixture (g)
$M_{cc,i}$	Mass of current collector per superficial area (g/cm^2)
$M_{chemical}$	Total chemical mass in a unit cell (g)
$M_{current\ collector}$	Total mass of current collectors in a unit cell (g)
M_e	Mass of electrolyte solution (g)
$M_{s,i}$	Mass of separators per superficial area (g/cm^2)
$M_{separator}$	Total mass of separators in a unit cell (g)
M_{total}	Total mass of a unit cell (g)
$N_{s,i}$	Number of layers per separator
$\rho_{a,i}$	Density of each material in anode (g/cm^3)
$\rho_{c,i}$	Density of each material in cathode (g/cm^3)
ρ_e	Density of electrolyte (g/mL)
R_{EC}	Electrolyte volume to capacity ratio (mL/mAh)
S_a	Specific capacity of anode (mAh/g)
S_c	Specific capacity of cathode (mAh/g)
T_c	Thickness of the cathode (cm)

$T_{s,i}$	Thickness of each separator (cm)
$V_{chemical}$	Total volume of chemicals used in a unit cell (cm^3)
$V_{CC,i}$	Volume of current collector per superficial area (cm^3/cm^2)
$V_{current\ collector}$	Total volume of current collectors in a unit cell (cm^3)
$V_{Electrolyte}$	Total volume of electrolyte (cm^3)
$V_{separator}$	Total volume of separators in a unit cell (cm^3)
V_{total}	Total volume of a unit cell (cm^3)

Energy Density and Capacity Cost Calculation

A unit cell consisting of one anode and one cathode was considered in this model. The cathode was treated as the limiting electrode, and the cathode mass was calculated from the battery capacity using the following relationship.

$$F_{c,CuO}M_cC_cD_c = C_T \quad \text{(S1)}$$

The anode mass was calculated using the following relationship:

$$F_{a,Zn}M_aS_aD_a = F_{c,CuO}M_cS_cD_c \quad \text{(S2)}$$

The volume of chemicals used in a unit cell was calculated using the following relationship (electrolyte volume was not considered for this calculation):

$$V_{chemical} = \frac{M_c}{(1-\varepsilon_c)}\sum_i \frac{F_{c,i}}{\rho_{c,i}} + \frac{M_a}{(1-\varepsilon_a)}\sum_i \frac{F_{a,i}}{\rho_{a,i}} \quad \text{(S3)}$$

The volume occupied by the separators was calculated using the following relationship:

$$V_{separator} = A \sum_i T_{s,i} \times N_{s,i} \quad \text{(S4)}$$

The volume occupied by the current collectors was calculated using the following relationship:

$$V_{current\ collector} = A \sum_i V_{cc,i} \quad \text{(S5)}$$

The volume of electrolyte was calculated using the following relationship:

$$V_{Electrolyte} = \varepsilon_c \times \frac{M_c}{(1-\varepsilon_c)}\sum_i \frac{F_{c,i}}{\rho_{c,i}} + \varepsilon_a \times \frac{M_a}{(1-\varepsilon_a)}\sum_i \frac{F_{a,i}}{\rho_{a,i}} + \varepsilon_s \times V_{separator} \quad \text{(S6)}$$

The total volume of the unit cell was calculated using the following relation:

$$V_{total} = V_{chemical} + V_{separator} + V_{current\ collector} \tag{S7}$$

The volumetric energy density of a unit cell was calculated as follows:

$$ED_V = \frac{C_T \times E \times 10^6}{V_{total}} \tag{S8}$$

The total mass of chemicals in a unit cell is given by the following equation:

$$M_{chemical} = M_a + M_c + M_e \tag{S9}$$

The total mass of separators in a unit cell was calculated using the following equation:

$$M_{separator} = A \sum_i M_{s,i} \times N_{s,i} \tag{S10}$$

The total mass of current collectors in a unit cell was calculated using the following equation:

$$M_{current\ collector} = A \sum_i M_{cc,i} \tag{S11}$$

The total electrolyte mass in a unit cell was calculated as follows:

$$M_e = \rho_e \times V_{Electrolyte} \tag{S12}$$

The total mass of a unit cell was calculated using the following relation:

$$M_{total} = M_{chemical} + M_{separator} + M_{current\ collector} \tag{S13}$$

The gravimetric energy density of a unit cell was calculated as follows:

$$ED_g = \frac{C_T \times E}{M_{total}} \tag{S14}$$

The areal loading of the cathode was calculated as follows:

$$L_c = \frac{M_c \times S_c \times F_{c,CuO}}{A} \tag{S15}$$

The thickness of the cathode was calculated as follows:

$$T_c = \frac{M_c}{(1-\varepsilon_c) \times A} \sum_i \frac{F_{c,i}}{\rho_{c,i}} \tag{S16}$$

The total chemical cost for the anode and cathode in a unit cell was calculated using the following equation (water was not included in the cost analysis):

$$C_{chemical} = M_c \sum_i F_{c,i} \times C_{c,i} + M_a \sum_i F_{a,i} \times C_{a,i} + M_e \times C_e \quad \text{(S17)}$$

The total separator cost was calculated as:

$$C_{separator} = A \sum_i C_{s,i} \times N_{s,i} \quad \text{(S18)}$$

The total cost of the current collectors was calculated using the following equation (one tab was accounted for each electrode, and tab cost was not area-based):

$$C_{current\ collector} = A \sum_i M_{cc,i} C_{mesh,i} + 2C_{tab} \quad \text{(S19)}$$

The total cost of a unit cell was then calculated using the following equation:

$$C_{unit\ cell} = C_{chemical} + C_{separator} + C_{current\ collector} \quad \text{(S20)}$$

The energy storage cost was calculated using the following equation:

$$C_{storage} = \frac{C_{unit\ cell} \times 10^6}{C_T \times E} \quad \text{(S21)}$$

The electrolyte volume to capacity ratio is calculated using the following equation:

$$R_{EC} = \frac{V_{Electrolyte}}{C_T} \quad \text{(S22)}$$

Supplemental References

(S1) Schorr, N. B.; Arnot, D. J.; Bruck, A. M.; Duay, J.; Kelly, M.; Habing, R. L.; Ricketts, L. S.; Vigil, J. A.; Gallaway, J. W.; Lambert, T. N. Rechargeable alkaline zinc/copper oxide batteries. *ACS Applied Energy Materials* **2021**, *4* (7), 7073-7082.

(S2) Arnot, D. J.; Schorr, N. B.; Kolesnichenko, I. V.; Lambert, T. N. Rechargeable alkaline Zn–Cu batteries enabled by carbon coated Cu/Bi particles. *Journal of Power Sources* **2022**, *529*, 231168.

(S3) Intratec. *Copper Oxide Price*. 2025. https://www.intratec.us/solutions/primary-commodity-prices/commodity/copper-oxide-prices (accessed 2026 03/18/2026).

(S4) ChemAnalyst. *Graphite Prices, Trend and Forecast*. https://www.chemanalyst.com/Pricing-data/graphite-1433 (accessed 2026 03/18/2026).

(S5) Made-in-China.com. *Bismuth Oxide, 2025 Manufacturers & Suppliers*. https://www.made-in-china.com/products-search/hot-china-products/Bismuth_Oxide_Price.html (accessed 2026 03/18/2026).

(S6) Intratec. *PTFE Price*. https://www.intratec.us/solutions/primary-commodity-prices/commodity/ptfe-prices (accessed 2026 03/18/2026).

(S7) Economics, T. *Zinc prices*. https://tradingeconomics.com/commodity/zinc (accessed 2026 03/18/2026).

(S8) Intratec. *Zinc Oxide Price*. https://www.intratec.us/solutions/primary-commodity-prices/commodity/zinc-oxide-prices (accessed 03/18/2026).

(S9) Charoentanaworakun, C.; Srisuriyachai, F.; Assabumrungrat, S.; Soottitantawat, A. Performance and salinity tolerance of palm oil-derived anionic biosurfactant and synthetic surfactant for waxy oil recovery in sandstone reservoirs. *Energy & Fuels* **2023**, *37* (17), 13191-13201.

(S10) Intratec. *Potassium Hydroxide Price*. https://www.intratec.us/solutions/primary-commodity-prices/commodity/potassium-hydroxide-prices (accessed 2026 03/18/2026).

(S11) Economics, T. *Copper Price*. https://tradingeconomics.com/commodity/copper (accessed 2026 03/18/2026).

(S12) Economics, T. *Nickel metal price*. https://tradingeconomics.com/commodity/nickel (accessed 2026 03/18/2026).
